# Repeated Recovery of *Staphylococcus saprophyticus* From the Urogenital Tracts of Women: Persistence Vs. Recurrence

**DOI:** 10.1155/S1064744995000056

**Published:** 1995

**Authors:** M. E. Rupp, J. Han, R. V. Goering

**Affiliations:** 1 Department of Internal Medicine University of Nebraska Medical Center 600 S. 42nd Street Omaha NE 68198-5400 USA; 2 Department of Medical Microbiology Creighton University School of Medicine Omaha NE USA

## Abstract

*Objective:* The purpose of this study was to determine whether colonization was persistent or recurrent in a small group of women who had repeated recovery of *Staphylococcus saprophyticus* from their urogenital tracts.

*Methods:* Paired isolates of *S. saprophyticus* from each of the study subjects were genotypically typed by plasmid fingerprinting and comparison of chromosomal-DNA restriction fragment-length polymorphism patterns by field-inversion gel electrophoresis (FIGE) and contour-clamped homogenous electric-field (CHEF) electrophoresis.

*Results:* All isolates of *S. saprophyticus* from the study subjects were classified as genetically unique by each of the typing methods.

*Conclusions:* The subjects experienced recurrent colonization with different isolates of *S. saprophyticus.* These findings may have broader implications regarding the pathogenesis and recurrence of *S. saprophyticus* urinary-tract infection.


Infectious Diseases in Obstetrics and Gynecology 2:218–222 (1995)
© 1995 Wiley-Liss, Inc.

### Repeated Recovery of *Staphylococcus saprophyticus* From the Urogenital Tracts of Women: Persistence Vs. Recurrence

**M.E. Rupp, J. Han, and R.V. Goering**

*Department of Internal Medicine, University of Nebraska Medical Center (M.E.R., J.H.), and Department of Medical Microbiology, Creighton University School of Medicine (M.E.R., R.V.G.), Omaha, NE*

### Abstract

***Objective:*** **The purpose of this study was to determine whether colonization was persistent or recurrent in a small group of women who had repeated recovery of *Staphylococcus saprophyticus* from their urogenital tracts.**

***Methods:*** **Paired isolates of *S. saprophyticus* from each of the study subjects were genotypically typed by plasmid fingerprinting and comparison of chromosomal-DNA restriction fragment-length polymorphism patterns by field-inversion gel electrophoresis (FIGE) and contour-clamped homogenous electric-field (CHEF) electrophoresis.**

***Results:*** **All isolates of *S. saprophyticus* from the study subjects were classified as genetically unique by each of the typing methods.**

***Conclusions:*** **The subjects experienced recurrent colonization with different isolates of *S. saprophyticus*. These findings may have broader implications regarding the pathogenesis and recurrence of *S. saprophyticus* urinary-tract infection.** © 1995 Wiley-Liss, Inc.

Key words

Urinary-tract infection, field-inversion gel electrophoresis, contour-clamped homogenous electric-field electrophoresis

*Staphylococcus saprophyticus* is second only to *Escherichia coli* as a cause of urinary-tract infection (UTI) in young women.[1–3] Investigators in the United States and Europe have demonstrated that *S. saprophyticus* causes up to 42% of UTIs in this population.[1–3] Infection with *S. saprophyticus* frequently involves the upper urinary tract and recurrence is not unusual.[1–3] A UTI with gram-negative enteric bacilli is often preceded by periurethral colonization from a fecal reservoir. In addition, it has been shown that women who experience repeated UTIs due to gram-negative organisms generally have recurrent infections with different strains of bacteria, rather than recurrent infections with the same strain.[4] In contrast, the pathogenesis of *S. saprophyticus* UTI is less clear. Some investigators have been unable to recover *S. saprophyticus* from mucosal sites, whereas others have stated that colonization of the periurethral membranes correlates well with infection. The persistence or recurrence of colonization has not been studied. In a previous study, we demonstrated that approximately 7% of asymptomatic women were colonized by *S. saprophyticus*.[5] Repeated recovery of *S. saprophyti-*

Address correspondence/reprint requests to Dr. Mark E. Rupp, Department of Internal Medicine, University of Nebraska Medical Center, 600 S. 42nd Street, Omaha, NE 68198-5400.

Presented at the 1993 Infectious Diseases Society of America Annual Meeting, New Orleans, LA, October 1993.

Received September 23, 1994
Accepted January 13, 1995

*cus* was observed in a smaller number of subjects. This study was undertaken to ascertain whether these women experienced persistent colonization of the urogenital and gastrointestinal tracts with the same strain of bacteria vs. recurrent colonization with different strains of *S. saprophyticus*.

### SUBJECTS AND METHODS

### Patients

In a previously published study designed to identify women colonized with *S. saprophyticus*, 19 of the 257 women studied were found to harbor *S. saprophyticus* in their urogenital tracts.[5] The subjects were identified prospectively from a population of women presenting for routine gynecologic care in an office-based gynecology practice. Of the 19 colonized women, 4 were recultured up to 4 times during the 12-month study. Three of these subjects had *S. saprophyticus* recovered on at least 2 follow-up visits and make up the study population for this report.

### Bacteria

Bacterial isolates were Gram-stained and tested for the production of catalase and coagulase. Coagulase-negative staphylococci were identified to species level, as described by Kloos and Schleifer.[6] Briefly, coagulase-negative staphylococci resistant to 5 μg of novobiocin/ml were classified as *S. saprophyticus* if they were urease positive; produced acid aerobically from maltose, sucrose, and trehalose; and did not produce acid from xylose.

### Preparation of Plasmid DNA

Plasmid DNA was purified by the modified use of a commercial kit (Magic™ Miniprep, Promega Corp., Madison, WI). Briefly, the isolates of *S. saprophyticus* were incubated overnight on trypticase soy agar (Bectin Dickinson, Baltimore, MD) plates at 37°C. The bacteria were harvested and suspended in 300 μl of TES (50 μM Tris, 5 μM EDTA, 50 mM NaCl) buffer. Fifty microliters of lysostaphin (Sigma Chemical Co., St. Louis, MO) solution (1 mg/ml) was added and the bacteria/lysostaphin solution was incubated overnight at 37°C without agitation to achieve lysis of the cells. Then, 300 μl of lysis solution (0.2 M NaOH and 1% sodium dodecyl sulfate) was added and 300 μl of neutralization solution (2.55 M potassium acetate, pH 4.8) was added. The tubes were inverted several times and the cellular debris was removed by centrifugation. The supernatant was treated with DNA purification resin (Promega Corp.) and the resin/DNA mixture was injected into the minicolumn according to the manufacturer's instructions. The column was washed with buffer containing 200 mM of NaCl, 20 mM of Tris-HCL, and 5 mM of EDTA. Next, the minicolumn was centrifuged in a microcentrifuge tube to dry the resin, and the DNA was eluted from the minicolumn with 50 μl of TE (10 μM Tris-HCl (pH 7.5), 1 mM EDTA) buffer. The plasmid preparations were analyzed by electrophoresis on 0.7% agarose gels.

### Preparation of Chromosomal DNA

The chromosomal DNA was prepared by a modification of the procedure reported by Goering and Winters.[7] Briefly, *S. saprophyticus* isolates were grown overnight at 37°C with shaking in 5-ml volumes of trypticase soy broth (Bectin Dickinson). A 1.0-ml aliquot of cells was transferred to a microcentrifuge tube, harvested by centrifugation, washed with 1.0 ml of TEN buffer [0.1 M Tris (pH 7.5), 0.15 M NaCl, 0.1 M EDTA; Sigma Chemical Co.], and resuspended in 0.5 ml of EC buffer [6 mM Tris HCl (pH 7.6), 1 M NaCl, 100 mM EDTA (pH 7.5), 0.5% Brij-58, 0.2% deoxycholate, 0.5% sodium lauroyl sarcosine]. A 0.5-ml aliquot of 2.0% sea-plaque agarose (FMC Corp., Rockland, ME) in EC buffer was added to the cell mixture, followed by 50 μl of 1 mg/ml lysostaphin solution (Applied Microbiology, Inc.). This suspension was quickly mixed and then cast into 10 × 5 × 1 mm blocks. The blocks were incubated at 37°C in EC buffer until clearing was observed, signifying lysis of the cells. The blocks were collected, suspended in TE buffer, and incubated for 1 h at 55°C. The blocks were then transferred to fresh TE buffer and stored at 4°C.

### Restriction Endonuclease Digestion and Analysis by Field-Inversion Gel Electrophoresis (FIGE) and Contour-Clamped Homogenous Electric-Field (CHEF) Electrophoresis

Restriction endonuclease digestion was performed by digesting a single agarose block with 20 U of *Sma*I in 250 μl of restriction buffer at 27°C for 2 h. The chromosomal restriction fragment-length polymorphism (RFLP) patterns were analyzed by

FIGE by using 0.8% agarose minigels in 0.5 × TBE buffer. Two switching patterns were used. For restriction fragments > 50 kb in size, initial 1.2 and 0.4 forward and reverse pulses were linearly increased over 3 h to 12 and 4 s, respectively, followed by 0.75 and 0.25 s forward and reverse pulses, respectively, for 30 min. Restriction fragments < 50 kb in size were separated for 0.4 and 0.2 s forward and reverse pulses, respectively, over a 3.25-h period. The RFLP patterns were also analyzed by CHEF gel electrophoresis on 14 × 20 cm agarose gels (0.8% SeaKem HGT; FMC Corp.) in 0.5x TBE buffer by using a CHEF-DR III pulsed-field elcetrophoresis system (Bio-Rad, Richmond, CA) at 6 V/cm, 14°C, with switching from 1 s to 30 s at a 120° angle for 22 h.

### RESULTS

### Patient Characteristics

The average age of the 3 persistently colonized women was 29 years. Two of the subjects were black, while the remaining subject was white. All 3 subjects reported a previous UTI, but none had experienced a symptomatic UTI in the preceding year. The paired *S. saprophyticus* isolates were recovered an average of 84 days apart. The rectum was colonized in all of the 3 repeatedly culture-postive women. In addition, 1 subject had *S. saprophyticus* recovered from both the urine and rectum on one of the visits. None of the women had urinary-tract symptoms, experienced a UTI during the 12-month period of observation, or received antibiotics.

### Plasmid DNA Analysis

The plasmid pattern observed for each of the pairs of isolates is shown in Figure 1. Each of the isolates exhibited a distinct plasmid pattern. The number of plasmid bands ranged from 1 to 7.

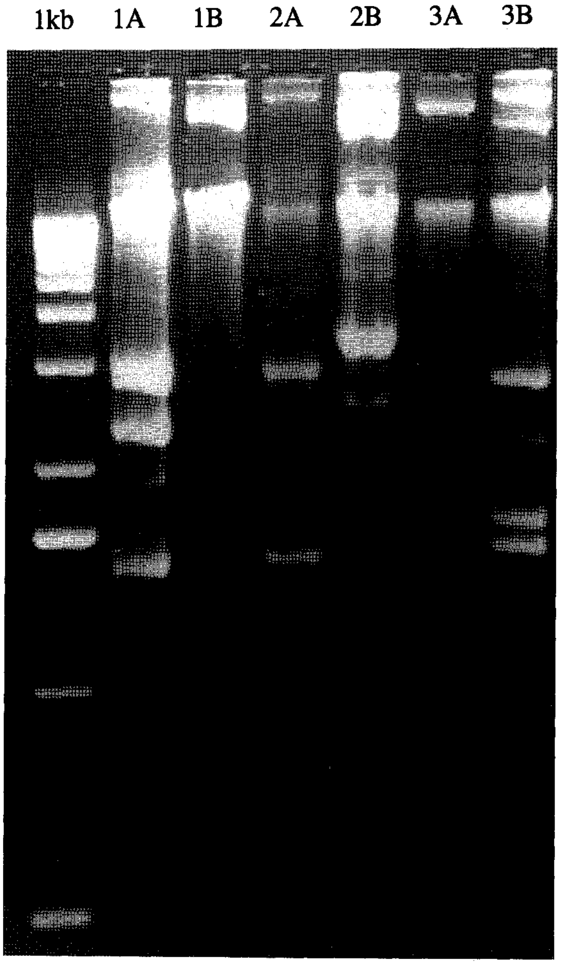

**Fig. 1.** Agarose gel electrophoresis of *S. saprophyticus* plasmid DNA (lane 1 molecular size standard is 1 kb: 0.5, 1.0, 1.6, 2.0, 3.0, 4.0, 5.0, 6.1 kb, respectively, from bottom). Numbers 1–3 (lanes 2–7) refer to patients 1–3; the letter A refers to the initial isolate and B refers to the subsequent isolate.

### Chromosomal DNA Analysis

The chromosomal DNA RFLP analysis is demonstrated by CHEF in Figure 2. Each isolate appeared to have a distinct pattern with a number of dissimilar bands between paired isolates. FIGE (not shown) also revealed distinct differences between the paired isolates.

### DISCUSSION

*S. saprophyticus* has been demonstrated to be an important uropathogen, second only to *E. coli* as a cause of acute UTI in young women. Several investigators have observed recurrence rates of *S. saprophyticus* UTI, despite appropriate therapy, as high as 30%.[1–3] Despite the frequency of *S. saprophyticus* UTI, and its high rate of recurrence, relatively little is known about the pathogenesis or epidemiology of these infections. Our observations may shed some light upon these questions. Our previous study supported the hypothesis that the pathogenesis of *S. saprophyticus* UTI is similar to that of enteric gram-negative organisms. Namely, uropathogenic organisms gain access to the female urinary tract from a fecal reservoir. It is known that recurrent UTIs due to gram-negative bacilli are most frequently due to recurrent infections with

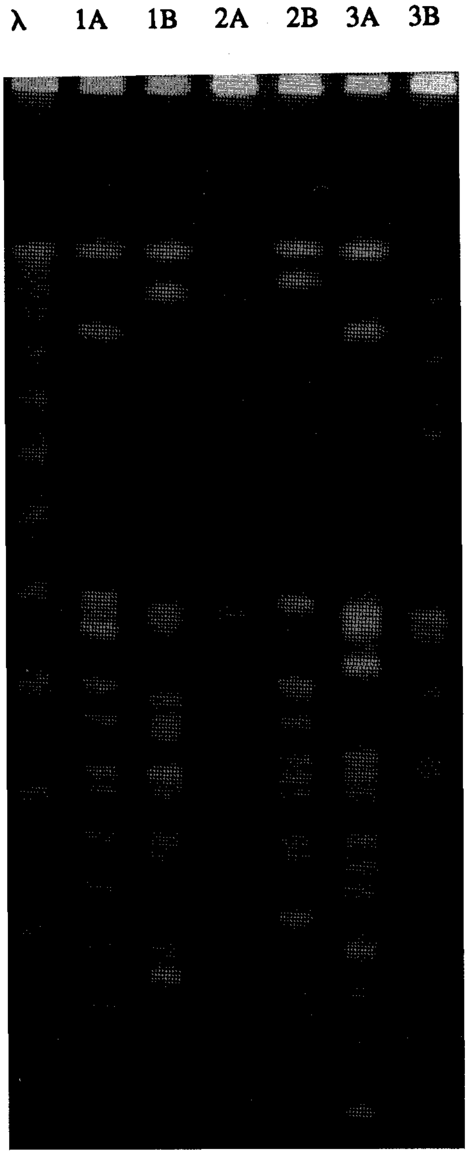

Fig. 2. CHEF gel electrophoresis of *Sma*I-digested chromosomal DNA from *S. saprophyticus* [lane 1 molecular size standard is lambda(48.5 kb) oligomers]. Numbers 1–3 (lanes 2–7) refer to patients 1–3; the letter A refers to the initial isolate and B refers to the subsequent isolate.

different strains of bacteria. Although the rate of recurrence of *S. saprophyticus* UTI is high, it is not known whether these recurrent infections are due to the persistence of the same strain or an acquisition of a different strain. This study indicates that women with repeated recovery of *S. saprophyticus* from their urogenital tracts experience recurrent colonization with different strains of *S. saprophyticus*. It is tempting to speculate that recurrent infections are also due to recurrent acquisitions of different strains of *S. saprophyticus*. However, as the subjects in our study did not experience a symptomatic UTI, this cannot be conclusively stated. The repeated recovery of *S. saprophyticus* in 3 of the 4 colonized subjects in whom it was sought suggests that it is a relatively common phenomenon in the small subset of women (approximately 7%) colonized by *S. saprophyticus*. It also suggests that this subset of women may have some predisposing condition for colonization that, to date, remains undefined.

Of additional interest is our use of molecular typing methods to examine questions of pathogenesis and epidemiology. This report is the first to use molecular typing methods to answer questions regarding the pathogenesis of *S. saprophyticus* colonization. Through use of these techniques, we were able to conclusively demonstrate the diversity of the strains and prove recurrent colonization with different strains of bacteria, which is particularly important in cases of recurrent infection for which treatment failure is considered and recommendations for longer courses of therapy are entertained.

From these data, we made the following conclusions: 1) molecular typing techniques are valuable tools in the analysis of the epidemiology and pathogenesis of *S. saprophyticus* colonization and infection; 2) *S. saprophyticus* can be repeatedly recovered from the urogenital/gastrointestinal tracts of a small proportion of asymptomatic women; and 3) although a sampling error cannot be excluded, these data indicate that these subjects experienced recurrent colonization with different strains of *S. saprophyticus* rather than persistent colonization with the same strain. In this regard, it is interesting to note that investigators in Sweden have recovered *S. saprophyticus* from a variety of foods,[8] which may serve as the route by which the gastrointestinal tracts of these women are recurrently colonized. There are some limitations of this study. First, a sampling error cannot be excluded, as only single colonies were chosen for storage and further evaluation in the first study. It is possible, therefore, that the subjects were colonized with several strains of *S. saprophyticus* and, due to our method of selecting only single colonies for evaluation, we have introduced a bias. Second, none of the women developed a symptomatic UTI. Therefore, we may be inaccurately extrapolating from colonization to infection.

Third, it is difficult to draw any final conclusions from a small group of patients.

### ACKNOWLEDGMENTS

This work was supported in part by a Young Investigator Award from the National Foundation for Infectious Diseases and a Grant-in-Aid from the American Heart Association, Nebraska Affiliate 9306247S.

### REFERENCES

1. Hovelius B, Mardh PA, Bygren P: Urinary tract infections caused by *Staphylococcus saprophyticus*: Recurrences and complications. J Urol 122:645–647, 1979.
2. Latham RH, Running K, Stamm WE: Urinary tract infections in young adult women caused by *Staphylococcus saprophyticus*. JAMA 250:3063–3066, 1983.
3. Wallmark G, Arremark I, Telander B: *Staphylococcus saprophyticus:* A frequent cause of acute urinary tract infection among female outpatients. J Infect Dis 138:791–797, 1978.
4. Komaroff AL: Acute dysuria in women. N Engl J Med 310:368–375, 1984.
5. Rupp ME, Soper DE, Archer GL: Colonization of the female genital tract with *Staphylococcus saprophyticus*. J Clin Microbiol 30:2975–2979, 1992.
6. Kloos WE, Schleifer KH: Simplified scheme for routine identification of human staphylococcal species. J Clin Microbiol 1:82–88, 1975.
7. Goering RV, Winters MA: A rapid method for the evaluation of chromosomal DNA from gram-positive cocci by field-inversion gel electrophoresis. J Clin Microbiol 30: 577–580, 1992.
8. Hedman P, Ringertz O, Eriksson B, Kvarnfors P, Andersson M, Bengtssonl, Olsson K: *Staphylococcus saprophyticus* found to be a common contaminant of food. J Infect 21:11–19, 1990.
